# Construction of an anchoring SSR marker genetic linkage map and detection of a sex-linked region in two dioecious populations of red bayberry

**DOI:** 10.1038/s41438-020-0276-6

**Published:** 2020-04-01

**Authors:** Yan Wang, Hui-Min Jia, Yu-Tong Shen, Hai-Bo Zhao, Qin-Song Yang, Chang-Qing Zhu, De-Li Sun, Guo-Yun Wang, Chao-Chao Zhou, Yun Jiao, Chun-Yan Chai, Li-Ju Yan, Xiong-Wei Li, Hui-Juan Jia, Zhong-Shan Gao

**Affiliations:** 1 Fruit Science Institute, College of Agriculture and Biotechnology, Zhejiang University, 310058 Hangzhou, China; 2 Shanghai Center for Plant Stress Biology, CAS Center for Excellence in Molecular Plant Sciences, Chinese Academy of Sciences, 201602 Shanghai, China; 3 Yuyao Forestry Technology Extension Center, 315400 Ningbo, China; 4 Institute of Forestry, Ningbo Academy of Agricultural Science, Ningbo, China; 5 Cixi Forestry Technology Extension Center, 315300 Cixi, China; 6 Linhai Forestry Technology Extension Center, 317000 Taizhou, China; 7 Forest & Fruit Tree Institute, Shanghai Academy of Agricultural Sciences, 201403 Shanghai, China

**Keywords:** Genetic markers, Agricultural genetics, Genetic hybridization

## Abstract

Red bayberry (*Morella rubra*) is an evergreen fruit tree found in southern China whose whole-genome sequence has recently been published. We updated the linkage map of the species by adding 118 SSR markers and the female-specific marker MrFT2_BD-SEX. The integrated map included eight linkage groups and spanned 491 cM. Eleven sex-associated markers were identified, six of which were located in linkage group 8, in agreement with the previously reported location of the sex-determining region. The MrFT2_BD-SEX marker was genotyped in 203 cultivated accessions. Among the females of the accessions, we found two female-specific alleles, designated W-b (151 bp) and W-d (129 bp). We previously found that **‘**Dongkui**’**, a female cultivar, could produce viable pollen (we refer to such plants **‘**Dongkui-male**’**) and serve as the paternal parent in crosses. The genotypes of the MrFT2_BD-SEX marker were W-b/Z in **‘**Biqi**’** and W-d/Z in **‘**Dongkui-male**’**. The progeny of a cross between these parents produced a 3:1 female (W-) to male (ZZ) ratio and the expected 1:1:1:1 ratio of W-b/W-d: W-b/Z: W-d/Z: Z/Z. In addition, the flowering and fruiting phenotypes of all the F1 progeny fit their genotypes. Our results confirm the existence of ZW sex determination and show that the female phenotype is controlled by a single dominant locus (W) in a small genomic region (59 kb and less than 3.3 cM). Furthermore, we have produced a homozygous “super female” (WW) that should produce all-female offspring in the F2 generation, providing a foundation for commercial use and presenting great potential for use in modern breeding programs.

## Introduction

Sexual systems in flowering plants are diverse, and ~6% of species are dioecious, with separate female and male plants^1^. Sex is often genetically determined, sometimes by sex chromosomes^2^. Sex chromosomes are typically thought to evolve from specific pairs of autosomes that may subsequently increase or decrease in size to form homomorphic or heteromorphic sex chromosomes^3^. Unlike the sex chromosomes of many animals, plants usually have homomorphic sex chromosomes that are not cytologically distinguishable in early stages of sex chromosome evolution, as demonstrated in kiwifruit (*Actinidia chinensis*)^4^, wild strawberry (*Fragaria virginiana*)^5^, willow (*Salix viminalis*)^6^, and garden asparagus (*Asparagus officinalis*)^7^. They can be divided into XY systems (homozygous XX females and heterozygous XY males), as observed in papaya (*Carica papaya*)^8^, persimmon (*Diospyros lotus*)^9^, date palm (*Phoenix dactylifera*)^10^, and poplar (*Populus trichocarpa*)^11^, and ZW systems (females heterozygous ZW and male homozygous ZZ), as observed in willow (*Salix viminalis*)^12^ and wild strawberry (*Fragaria virginiana*)^5^.

In several plants with homomorphic sex chromosomes, it has been reported that sex determination is controlled by a small region that prevents recombination around the sex-determining locus. For example, wild strawberry (*Fragaria virginiana*) has two sex-determining loci, showing recessive male sterility (*g*) and dominant female fertility (*A*), with a 280 kb sex determination region located in linkage group 6^5^. In asparagus (*Asparagus officinalis*), sex determination is controlled by a single locus, M, on chromosome 5, including two tightly linked genes: the female suppressor (*F*) and male activator (*M*) ^13^.

Sex determination in plants is a universal biological process and is very important in agriculture, horticulture, and environmental protection^14^, because female and male plants present different value, especially in early stage identification in perennial species. Red bayberry (*Morella rubra*, formerly *Myrica rubra*) is usually dioecious, with female plants being used for commercial fruit production and males for pollination. However, it has a long juvenile period, making it time-consuming to identify the plants**’** sexes. As red bayberry is an important fruit in southern China, it is important to study the sex-determination mechanism of this species. Using the assembled genomes of one female and one male individual, we previously proposed a ZW model of sex determination and identified a small (59 Kb) female-specific region at the distal end of chromosome 8^15^. However, the ZW model has not been verified, and how the sex-linked region is inherited remains largely unknown.

**‘**Biqi**’** and **‘**Dongkui**’** are two economically important, genetically distinct female red bayberry cultivars^16,17^. In 2011, we identified rare male catkins and viable pollen in the **‘**Dongkui**’** cultivar after applying a vigor control regulator (containing uniconazole) to plants in July and August^18^ and designated the source pollen as **‘**Dongkui-male**’**. F1 progeny were successfully obtained from the first cross between **‘**Biqi**’** and **‘**Dongkui-male**’**^19^.

The availability of F1 progeny allowed us to add codominant SSR markers to the SNP genetic linkage map used for genome assembly and genetic analysis. Segregation in the F1 population revealed a dominant female-specific W-locus controlling sex determination, confirming the ZW sex chromosome system of red bayberry. The F1 population also produced homozygous “super female” (WW) individuals, which could theoretically generate all-female offspring in F2 for further study and commercial breeding.

## Results

### Linkage map construction

To construct a genetic linkage map, 95 F1 progeny and 127 SSR markers that were heterozygous in **‘**Biqi**’**, **‘**Dongkui-male**’** or both were selected. The MrFT2_BD-SEX marker, which was developed from the female-specific region^15^ and exhibited different fragments between the parents **‘**Biqi**’** and **‘**Dongkui-male**’**, was also included. Based on the published biparental genetic linkage map of red bayberry^15^, we then estimated a new integrated high-density SSR-SNP linkage map (Fig. S1) with 3,073 SNP and 118 SSR markers and MrFT2_BD-SEX, while the nine unmapped SSR markers presented unclear genotypes or distorted segregation ratios. The final map spanned 491 cM with an average marker interval of 0.15 cM (Table 1).

**Table 1:** Summary of the integrated linkage groups of the ‘Biqi’ x ‘Dongkui’ mapping population

Linkage group	Number of markers	Map length (cM)	Average marker distance (cM)
LG1	485	79.75	0.16
LG2	435	61.91	0.14
LG3	401	53.52	0.13
LG4	387	53.55	0.14
LG5	359	55.47	0.15
LG6	354	74.49	0.21
LG7	412	52.32	0.13
LG8	358	59.84	0.17
Total	3191	490.85	0.15

**Fig. 1:**
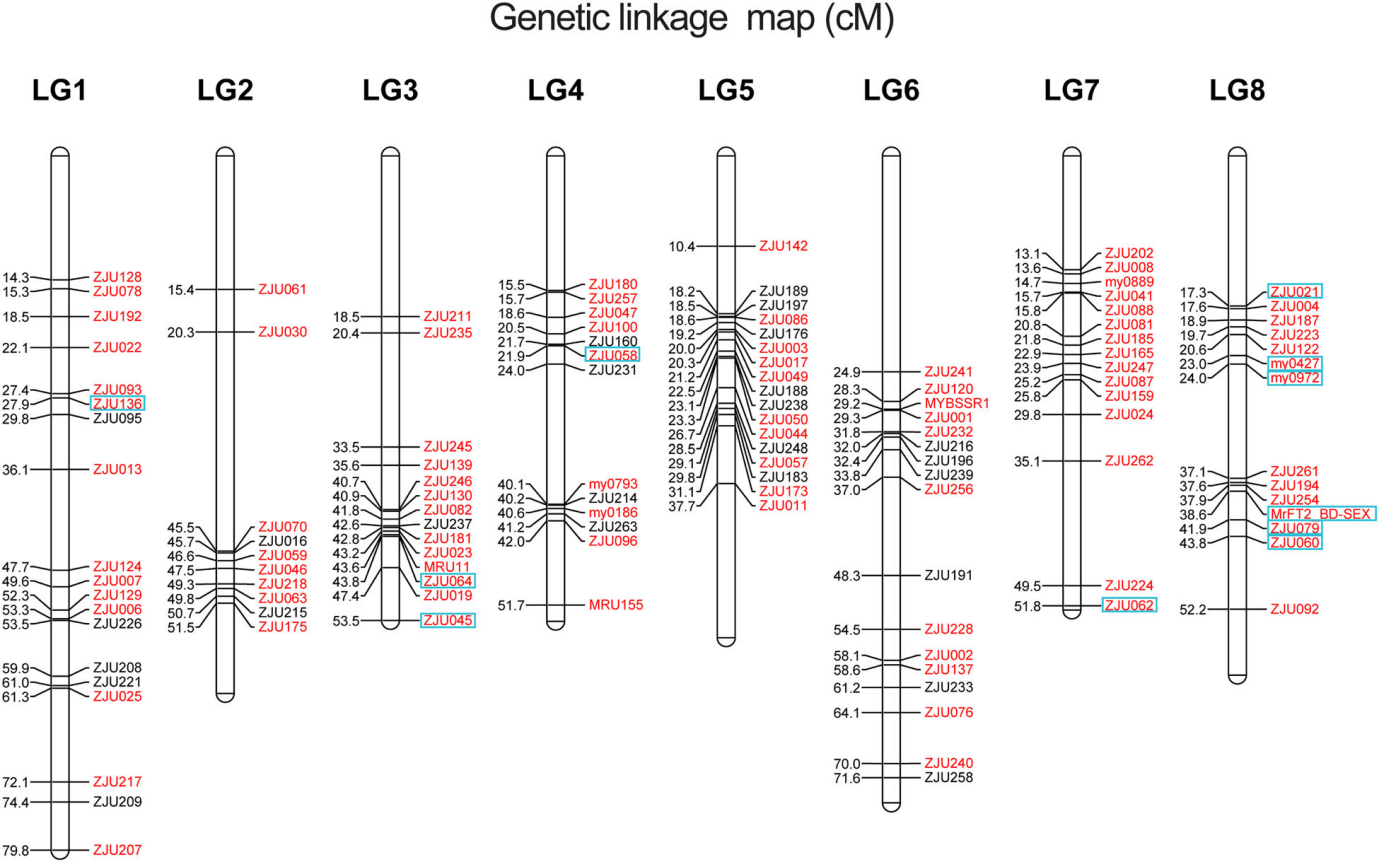
Integrated SSR genetic linkage map of red bayberry (*Morella rubra*). Eight linkage groups (LG) with 118 SSR markers and MrFT2_BD-SEX. Ninety-four markers, indicated in red, were used to analyze the association between markers and sex traits in 203 accessions. The 11 boxed markers were identified as being related to the sex phenotype in cultivated populations

We screened all SSR markers from the high-density SSR-SNP map (Fig. S1) to obtain an SSR-based genetic linkage map (Fig. 1) containing 118 SSR markers and the MrFT2_BD-SEX marker. Among the mapped markers, 45 (37.8%) segregated in female meiosis (with the parental genotypes lm × ll, where different letters denote distinct alleles), and 25 (21.0%) segregated in male meiosis (nn × np), while 43 (36.1%) fully informative markers were heterozygous in both parents (12 with four distinct alleles, ab×cd type, and 31 with 3 alleles, ef × eg), and 6 (5.0%) were partially informative in both parents (hk × hk). Searching for markers near the female-specific marker MrFT2_BD-SEX yielded two flanking SSR markers, ZJU254 and ZJU079, at distances of 0.7 cM and 3.3 cM from the MrFT2_BD-SEX marker, respectively (Fig. 1). Two SSR markers that were previously predicated as sex-associated, ZJU062 and ZJU130^17^, were located in LG7 and LG3, respectively (Fig. 1).

We next obtained the physical linkage map by aligning the PCR product sequences of the mapped markers in an SSR-based genetic linkage map to the chromosome sequences (Fig. S2). One hundred and eleven markers were anchored to the assembled genome sequences, among which three were tandemly duplicated (ZJU247, ZJU238, and ZJU021), one was duplicated in a different region of the same linkage group (my0889), and two were duplicated in different linkage groups (ZJU202 and ZJU137). Among the remaining eight SSR markers, five matched unmapped scaffolds, and three did not match any sequences.

### SSR marker association analysis in the dioecious population

Based on the SSR-based linkage map, 93 SSR markers and MrFT2_BD-SEX were used to evaluate the association between the markers and the sex phenotype in 203 accessions, including 95 of the main cultivated varieties (females), 107 males, and one monoecious individual, collected from Zhejiang, Jiangsu, Fujian, Hunan, Guangxi, and Guizhou Provinces (Table S1). Twelve accessions (four females and eight males) from Hunan Province and 39 SSR markers were added compared with our earlier report^17^.

**Fig. 2:**
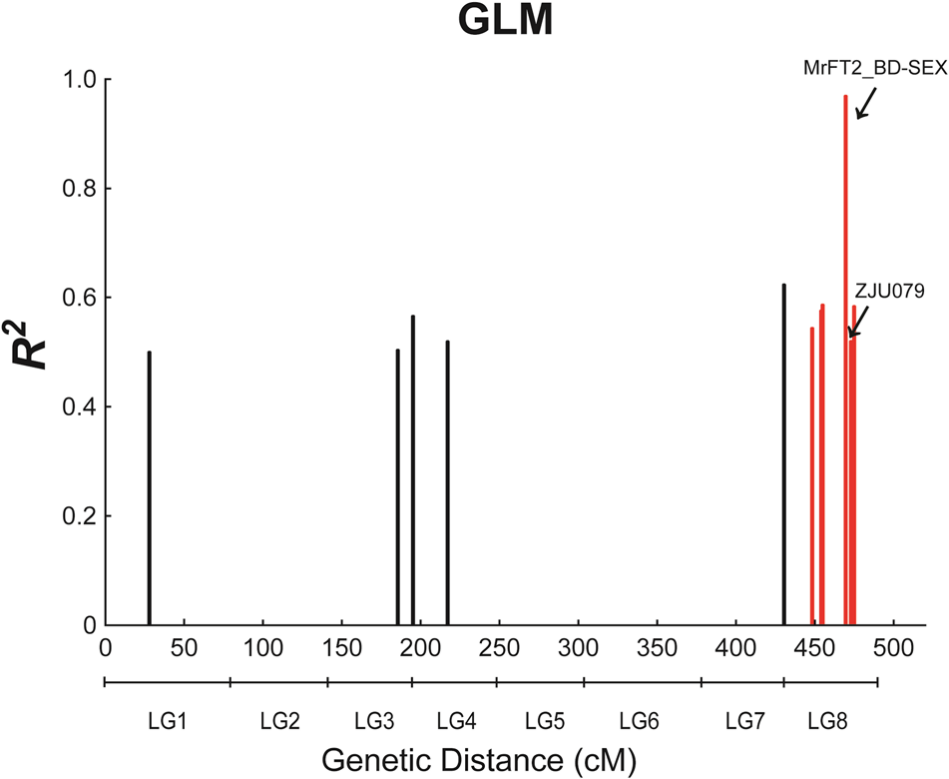
Positions of 11 sex-associated markers based on the general linear model. The total genetic distance, covering eight linkage groups, is indicated on the *X*-axis. The explained phenotypic variation between the markers and the sex population is shown on the *Y*-axis. The six potential sex-associated markers are indicated in red in LG8. The sex-associated markers are indicated by arrows

Structural analysis divided the 203 accessions into two groups, as the lnP (*K*) function increased without a turning point, while the delta *K* (Δ*K*) function presented a clear inflexion point at *K* = 2. We analyzed the correlation between the markers and sex traits by combining the population structure, genotype, and sex phenotype data using the general linear model (GLM) method. The results showed that 11 markers were associated with the sex phenotype, and six of them were located in LG8 (Table S2). The highest sex phenotype variation (*R*^*2*^) value, of 0.97, was obtained for the MrFT2_BD-SEX marker (Fig. 2 and Table 2); this marker was amplified only in the female accessions, generating products of 129 bp and 151 bp, with allele frequencies of 0.484 and 0.516, respectively (Table S2). The MrFT2_BD-SEX marker therefore accurately identifies the sexes of cultivated accessions, which implies that the marker is completely sex linked in the cultivated red bayberry accessions. Based on the population structure^17^, we found the 129 bp allele in most of the **‘**Fenhong**’** and **‘**Dongkui**’** subgroups and the 151 bp type in the **‘**Biqi**’** subgroup (Table S1) and the monoecious individual. For the ZJU079 marker, which was the marker closest to MrFT2_BD-SEX in linkage group 8 (3.3 cM) (Fig. 2 and Table 2), the most common alleles in males were 124 and 130 bp, versus 128 and 134 bp in females (Table S2).

**Table 2:** The 11 sex-associated markers based on general linear model (GLM) analysis

Markers	LG	Position (cM)	*R*^*2*^	p_M	p-P	p-adj
ZJU136	LG1	27.89	0.50	2.92E-09	1.00E-04	1.00E-04
ZJU064	LG3	43.82	0.50	8.86E-14	1.00E-04	1.00E-04
ZJU045	LG3	53.52	0.57	6.18E-11	1.00E-04	1.00E-04
ZJU058	LG4	21.9	0.52	2.10E-11	1.00E-04	1.00E-04
ZJU062	LG7	51.79	0.62	4.25E-27	1.00E-04	1.00E-04
ZJU021	LG8	17.33	0.54	1.12E-20	1.00E-04	1.00E-04
my0427	LG8	22.95	0.58	1.31E-17	1.00E-04	1.00E-04
my0972	LG8	23.95	0.59	1.49E-15	1.00E-04	1.00E-04
MrFT2_BD-SEX	LG8	38.55	0.97	0.00E + 00	1.00E-04	1.00E-04
ZJU079	LG8	41.92	0.52	8.84E-18	1.00E-04	1.00E-04
ZJU060	LG8	43.83	0.58	5.56E-24	1.00E-04	1.00E-04

The number of permutations is 50,000

*LG* linkage group, *Position* Genetic distance in linkage map, *R2* The portion of total variation explained by the markers only in this model, *p_M* the p-values of markers for F-tests, *p-P* The test of individual markers based on the permutations, *p-adj* The marker p-value adjusted for the permutation test and controlling the familywise error rate

### Sex phenotype in the F1 mapping population

**Fig. 3:**
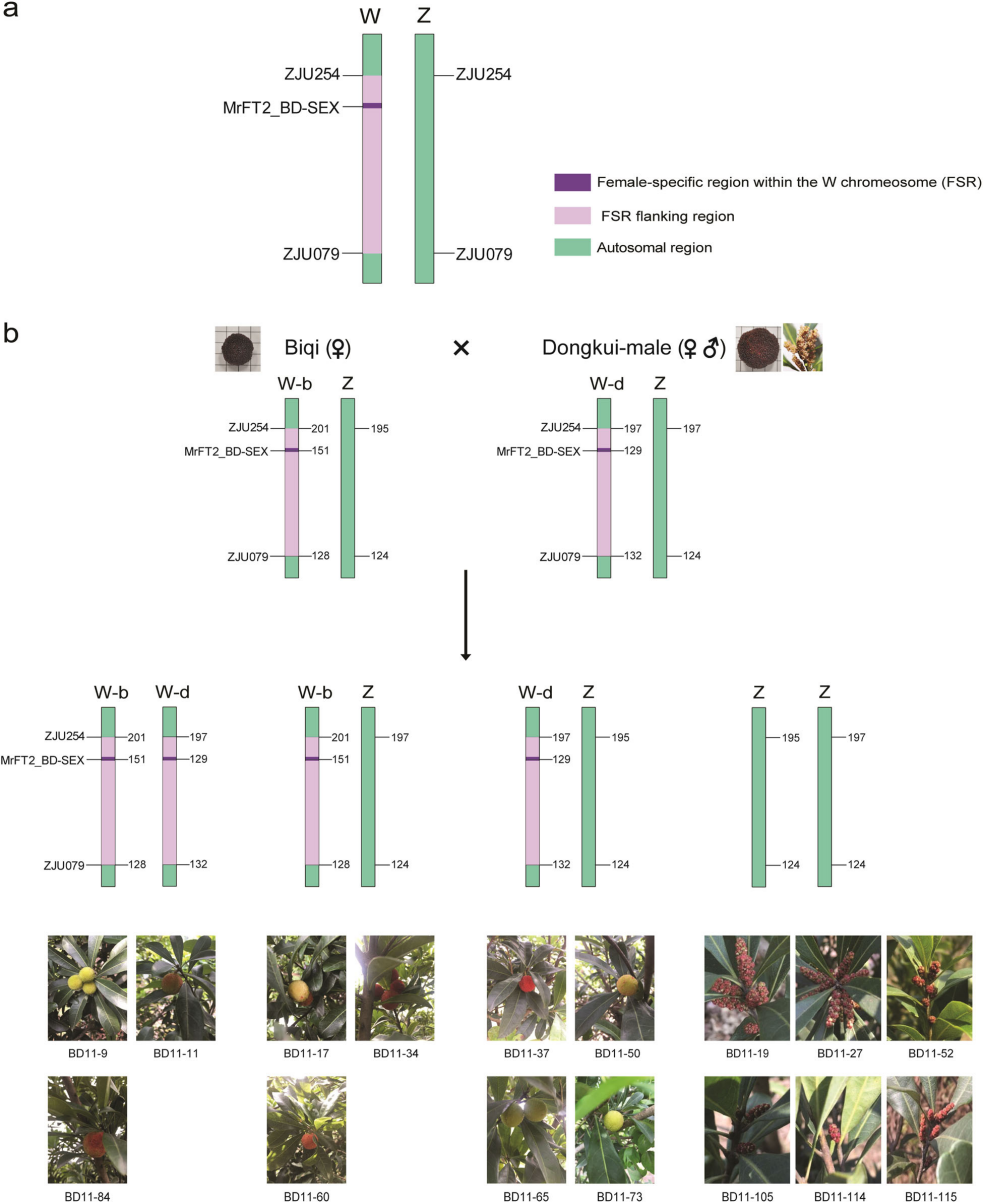
Sex-determination model and the sex phenotypes and genotypes of 16 F1 seedlings. **a** The sex-determination model of ZW sex chromosomes in red bayberry. The female-specific region (FSR) within the W chromosome is indicated in purple, the FSR flanking region is indicated in pink, and the autosomal region is indicated in green. **b** The genotypes of the parents are predicted to be W-b/Z (**‘**Biqi**’**) and W-d/Z (**‘**Dongkui-male**’**). The genotypes of the F1 progeny are segregated into four types: W-b/W-d, W-b/Z, W-d/Z, and Z/Z, and the corresponding individuals' sex phenotype are shown in photos

The F1 seedlings were planted in 2013 and 2015. Sixteen individuals flowered in 2019, and their sex phenotypes were determined from their catkins and fruits (Fig. 3b and Table S3). The F1 seedlings were also genotyped for the MrFT2_BD-SEX marker, which identified 70 individuals as females (129 bp, 151 bp, or heterozygous for both these alleles) and 25 as males (no amplification), in agreement with the Mendelian segregation ratio of 3:1 expected under the assumption that the female is heterozygous ZW (Chi-square *p* value 0.77). The sex phenotypes of the sixteen offspring that flowered were consistent with the MrFT2_BD-SEX amplification results (Table S3, Fig. 3b).

According to the ZW model, the genotypes of the **‘**Biqi**’** and **‘**Dongkui-male**’** parents at the MrFT2_BD-SEX marker are W-b/Z (151 bp) and W-d/Z (129 bp) (Fig. 3a), respectively. In the ZW × ZW cross population, we observed the expected four genotypes: (1) 151 bp alone (W-b/Z, 18 individuals); (2) 129 bp alone (W-d/Z, 33 individuals); (3) both 151 bp and 129 bp (W-b/W-d, 19 individuals); and (4) Z/Z males with no detectable marker allele (25 individuals). The numbers of individuals of the different genotypes fit the expected 1:1:1:1 ratio (Chi-square test *p* = 0.11 > 0.05). The viable W-b/W-d genotype indicated that the WW genotype could represent a “super female” population, which should be useful for producing all-female F2 progeny.

Around the sex marker MrFT2_BD-SEX, one flanking marker, ZJU079, was heterozygous in **‘**Biqi**’** (128/124 bp) and **‘**Dongkui-male**’** (132/124 bp), with 128 bp and 132 bp alleles in the W region (since they were transmitted to most female progeny) and a 124 bp allele in the Z region. The other flanking marker, ZJU254, was heterozygous in **‘**Biqi**’** (195/201 bp) but homozygous in **‘**Dongkui-male**’** (197/197 bp).

## Discussion

### The female phenotype of red bayberry is controlled by a dominant locus in linkage group 8

Understanding sex-determination mechanisms is essential to illustrate the evolution of sex chromosomes. Red bayberry is normally dioecious with a few monecious individuals, and it has homomorphic ZW sex chromosomes, as observed in wild strawberry^5^ and poplar^12^. In this study, the “super-female” genotype (homozygote WW) was viable in the F1 progeny, unlike the situation in papaya (*Carica papaya*), where the YY genotype is lethal^20^. To the best of our knowledge, the viability of WW genotypes was first reported in fruit trees. These results suggest that red bayberry represents stage two of sex chromosome evolution, with a small W-linked region that evolved recently enough for genetic degeneration not to have become advanced^1^. The WW genotype provides valuable material that may contribute to the assembly of the W region and the determination of the boundary between the Z- and W-linked regions of this chromosome. Homozygous “super female” (WW) plants will also be useful for further breeding programs with the aim of producing all-female F2 progeny for fruit production.

In the F1 progeny and 203 cultivated accessions, MrFT2_BD-SEX was inferred to be a completely sex-linked marker, as it accurately identified the sex of red bayberry. Based on the SSR-based linkage map (Fig. 1), two SSR markers flanking MrFT2_BD-SEX in the F1 seedlings showed that ZJU079 (3.3 cM) might be partially sex linked according to Mendelian segregation and the Chi-square test (*p* = 0.27), while ZJU254 (0.7 cM) might not be sex linked because of the homozygous fragment of **‘**Dongkui-male**’**, although it was very close to the sex marker MrFT2_BD-SEX (Table S3). This result is consistent with that of GLM analysis in the 203 cultivated accessions. In our study, the physical distance between MrFT2_BD-SEX and ZJU079 was found to be 1.0 Mb (Fig. S1), which is larger than the female-specific region (59 Kb) reported before^15^. This shows that ZJU079 is sex-related and narrows the female-specific region to less than 3.3 cM. This result is consistent with the homomorphic sex chromosomes of this plant, and it suggests that recombination has not been suppressed and occurs in genome regions close to the sex-determination locus^6^. The complete W-linked region of red bayberry chromosome 8 appears to be physically small (59 kb and less than 3.3 cM on chromosome 8), even smaller than the regions recently identified in several other dioecious plants, including willow (840 kb on chromosome 15)^6^, wild strawberry (280 kb in linkage group 6), and kiwifruit (1 Mb on chromosome 25)^21^.

In red bayberry, the genetic background of cultivated accessions is complex, with the female plants having been selected over many years for better fruit quality, while the male plants are basically wild accessions due to their low commercial value. Without linkage map information, the allele frequencies in the two sex populations identified two markers, ZJU130 and ZJU062, that were moderately associated with sex^17^, which have now been located in linkage groups 3 and 7, respectively, implying that they are accompanying sex stratification factors rather than sex-determining factors. In this study, GLM analysis identified ten SSR markers that might be associated with the sex phenotype, including ZJU062 from a previous report^17^. Five SSR markers were located in LG8, among which two (ZJU079 and ZJU060) were located very close to MrFT2_BD-SEX, and the other three (ZJU021, my0427, and my0972) were located at the beginning of LG8, which might be related to homologues of female-specific genes. Markers located in other linkage groups may be related to the evolution of other traits in male and female plants, such as flowering, variety selection and fruit quality.

It is interesting that the female cultivars occasionally exhibit mutated or induced male branches in nature. In our previous study, we found two types of sex-changing branches in red bayberry. One (**‘**Biqi-male**’**), from the **‘**Biqi**’** cultivar, was permanent, as some branches bore male flowers and remained unchanged in the following years; the other (**‘**Dongkui-male**’**) showed induced viable pollen in the **‘**Dongkui**’** cultivar and resulted in occasional male flowers on female branches, which returned to an all-female phenotype in the following years. For both of these types of sex-changing branches, the viable pollen presented the same genotype as the corresponding female individuals (Table S1) according to the MrFT2_BD-SEX marker. It is unknown why males can show amplification of the same genotypes as female individuals in red bayberry. We suggest that the ZW females might be controlled by two sex-determining genes within the young sex chromosome^22^ and might have undergone the loss of a W-linked male-suppressing factor, similar to the suppressor of feminization (*SyGI*) and maintenance of male (M) (*FrBy*) in kiwifruit, leading to a two-factor sex-determination system^23^. Similarly, mutant XY males in *Silene latifolia* have lost a female-suppressing factor and become functional hermaphrodites^24^. Considering the male-specific DNA methylation patterns in poplar^25^ and the role of microRNAs as the key sex regulators in persimmon^9^, it is also reasonable to speculate that epigenetic regulation mechanisms such as DNA methylation, histone modifications and microRNAs might also contribute to sex determination, in addition to genetic regulation in red bayberry.

### The genetic linkage map gives us a better understanding of sex determination in red bayberry

De novo genome sequencing has had a profound impact on genetics and various applications^26,27^. Red bayberry is a perennial woody plant with a long juvenile stage. In this study, we constructed an integrated SSR-SNP linkage map with an average marker distance of 0.15 cM. This SSR-SNP genetic map provides a powerful tool for locating sex-related markers and identifying the sex-determination mechanisms of cultivated accessions and cross populations. The map will also serve as a reference to align different mapping populations for genome assembly and lays the foundation for the genetic and genomic analysis of traits related to disease resistance, productivity and fruit quality. The mapped SSR markers can also be valuable for identifying cultivars, evaluating genetic diversity, and studying the relationships and origins of cultivars.

According to the physical map (Fig. S2), certain SSR markers were distributed in multiple linkage groups, which might be due to repeat sequences in the genome, as the red bayberry genome harbors 36.4% repetitive sequences^15^, indicating that duplications are not implausible. SSR duplication has been reported in other fruit, such as strawberry^28^, jujube^29^, pear^30^, and citrus^31^. The comparison of the SSR-based linkage map and physical map revealed some differences in the marker order, which may be due to the lack of one-to-one correspondence of the markers to the scaffolds^15^ and the high number of repetitive sequences affecting the quality of the assembly. The SSR markers matched unmapped scaffolds or did not match any sequences, probably because the anchored physical linkage map covered 90% of the assembled genome and 87% of the whole genome^15^. Some order issues remain, and 10% of the genome sequence needs to be corrected and assembled.

The cultivated female varieties were divided into two groups according to the MrFT2_M2-SEX marker, with the first consisting of the **‘**Biqi**’** series, while the second consists of the **‘**Dongkui**’** and **‘**Fenhong**’** cultivars (Table S1). This suggests that cultivated varieties might have evolved from two progenitors. The difference between the two genotypes was a difference in fragment size, with **‘**Biqi**’** presenting a larger fragment than **‘**Dongkui**’**. This indel could have important consequences for the evolution of plant genomes because variations in self-fertilization in plant species lead to differences in the heterozygosity of alleles^32^.

In conclusion, we developed a new SSR-SNP genetic linkage map for red bayberry. From the map, we identified a sex-related SSR marker, ZJU079, and confirmed that the sex-determination region is very short (59 kb and less than 3.3 cM) in linkage group 8. Our results confirm that the sex mechanism is genetically controlled by a single dominant female-specific locus (W).

## Materials and methods

### Plant materials and DNA extraction

An F1 population was derived from hybrid progeny of two cultivars, **‘**Biqi**’** and **‘**Dongkui-male**’**^19^, in 2011 and 2013. From this population, 95 samples were selected for mapping, including 77 individuals obtained from the 2011 crossing population reported previously^19^ and 18 generated in 2013. Young leaves of the hybrids were collected from Yuyao, Zhejiang Province, China, frozen in liquid nitrogen and stored at −40 °C.

Genomic DNA was extracted from these samples using the modified cetyltrimethylammonium bromide (CTAB) method^33^ and then diluted to 30 ng µl^−1^ for PCR amplification.

### SSR marker amplification

The SSR markers were developed from WGS sequences^16,34,35^ and the *M. rubra* EST database^36^. The origin of the polymorphic markers is given in Table S4. A three-primer strategy was used for the PCR amplification of SSR markers, involving a regular reverse primer, a forward primer with an M13 tail at the 5**’** end, and a forward primer including an M13 tail with one of the following fluorescence labels at the 5**’** end: FAM (blue), HEX (green), NED (yellow), or PET (red) (Invitrogen, Shanghai, China). The MrFT2_BD-SEX marker was developed from the DNA sequence of the female-specific putative gene *MrFT2*, and its fragment size differs between **‘**Biqi**’** and **‘**Dongkui-male**’**. The forward primer was FAM 5**′**-GCGGTATAGTAATCAGGATTCCAT-3**′**, while the reverse primer was 5**′**-GGGTTCCATCATAAGGACATTTGT-3**′**. We first chose two parents and six of their progeny to examine the polymorphism of SSR markers. Eight polymorphic SSR markers (Invitrogen, Shanghai, China) were selected for true hybrid identification^19^. The PCR system and procedure used for SSR marker analysis were as described by Terakawa et al.^34^ and Jiao et al.^16^. The 15 µl reaction mixtures contained 5 pmol reverse primer, 1 pmol M13-tailed forward primer, 4 pmol M13-labeled fluorescent primer, and *PremixTaq*^TM^ Hot Start Version (TaKaRa, Dalian, China) in an amount according to the manufacturer**’**s instructions, and 30 ng genomic DNA template. The two-step PCR amplification procedure was as follows: 94 °C for 5 min, 30 cycles of 30 s at 94 °C, 30 s at 58 °C, and 30 s at 72 °C, and then eight cycles of 30 s at 94 °C, 30 s at 53 °C, and 30 s at 72 °C, with a final extension at 72 °C for 10 min. The PCR mixture for MrFT2_BD-SEX was the same as for the SSR markers but without an M13-tailed forward primer, and the PCR amplification procedure was as follows: 94 °C for 5 min, 35 cycles of 30 s at 94 °C, 30 s at 58 °C, 30 s at 72 °C, and a final extension at 72 °C for 10 min. The PCR products with different sizes and fluorescence labels were mixed with the GeneScan LIZ500 standard (Applied Biosystems) and detected in an ABI3130 DNA analyzer with the allele size analyzed by GeneMapper v4.0 software (Applied Biosystems, CA).

### Linkage map construction

The genotype data of the polymorphic SSR markers and MrFT2_BD-SEX were scored by JoinMap 4.1^37^. The population type was CP, a population resulting from a cross between two heterozygous diploid parents and possibly an unknown linkage phase. The segregation type codes were ab × cd, ef × eg, hk × hk, lm × ll, and nn × np. According to the expected Mendelian ratio, the segregation of markers was tested to identify distorted markers according to the Chi-square test, and these markers were excluded. Markers were grouped using a threshold independence logarithm of odds (LOD) value of 7.0, and eight linkage groups were selected on the basis of the known chromosome number according to a cytological study in Myricaceae^38^, in accordance with the order of chromosomes published by Jia et al.^15^. The regression mapping algorithm and Kosambi**’**s mapping function^39^ were applied for marker ordering in the group, and the map distances were generated in centimorgans. MapChart^40^ was used to draw the linkage maps.

### General linear model analysis of two sex populations

STRUCTURE v.2.0^41^ was applied to 203 accessions to infer the population structure using a 100,000 times burn-in period and 100,000 MCMC iterations. The test *K* values ranged from 1 to 10 with ten independent runs. STRUCTURE HARVESTER (http://taylor0.biology.ucla.edu/structureHarvester/)^42^ was used to generate the Q-matrix under the most likely *K* value according to the delta *K* (Δ*K*) value. The associations between the genotypes and phenotypes of the 203 accessions were evaluated with the general linear model (GLM) using TASSEL version 2.1.0.

## Supplementary information

Supplemental Materials
