# Supplementary material for: Construction of an anchoring SSR marker genetic linkage map and detection of a sex-linked region in two dioecious populations of red bayberry
Source: Hortic Res. 2020 Apr 1;7:53. doi: 10.1038/s41438-020-0276-6 (PMC7109115; doi:10.1038/s41438-020-0276-6)
Supplement: Supplementary file 1 — Supplemental Materials [file 41438_2020_276_MOESM1_ESM.docx]

**Construction of anchoring SSR marker genetic linkage map and detection of a sex-linked region in two dioecious populations of red bayberry**

Yan Wang^1^, Hui-Min Jia^2*^, Yu-Tong Shen^1^, Hai-Bo Zhao^1^, Qin-Song Yang^1^, Chang-qing Zhu^1^, De-li Sun^1^, Guo-Yun Wang^3^, Chao-chao Zhou^3^, Yun Jiao^4^, Chun-Yan Chai^5^, Li-Ju Yan^6^, Xiong-wei Li^7^, Hui-Juan Jia^1^, Zhong-Shan Gao^1*^

^1^ Fruit Science Institute, College of Agriculture and Biotechnology, Zhejiang University, Hangzhou, 310058, China

^2^ Shanghai Center for Plant Stress Biology, CAS Center for Excellence in Molecular Plant Sciences, Chinese Academy of Sciences, Shanghai, 201602, China

^3^ Yuyao Forestry Technology Extension Center, Ningbo 315400, China

^4^ Institute of Forestry, Ningbo Academy of Agricultural Science, Ningbo, China

^5^ Cixi Forestry Technology Extension Center, 315300, China,

^6^ Linhai Forestry Technology Extension Center, 317000, China.

^7^ Forest & Fruit Tree Institute, Shanghai Academy of Agricultural Sciences, Shanghai, 201403, China

^*^Correspondence:

Professor Zhongshan Gao, email: [gaozhongshan@zju.edu.cn](mailto:gaozhongshan@zju.edu.cn), Tel: ++86-571-88982225; 13819479231 and Dr. Huimin Jia, email: jiahuimin1988@163.com

**Supplemental Materials**

**Figures**

**
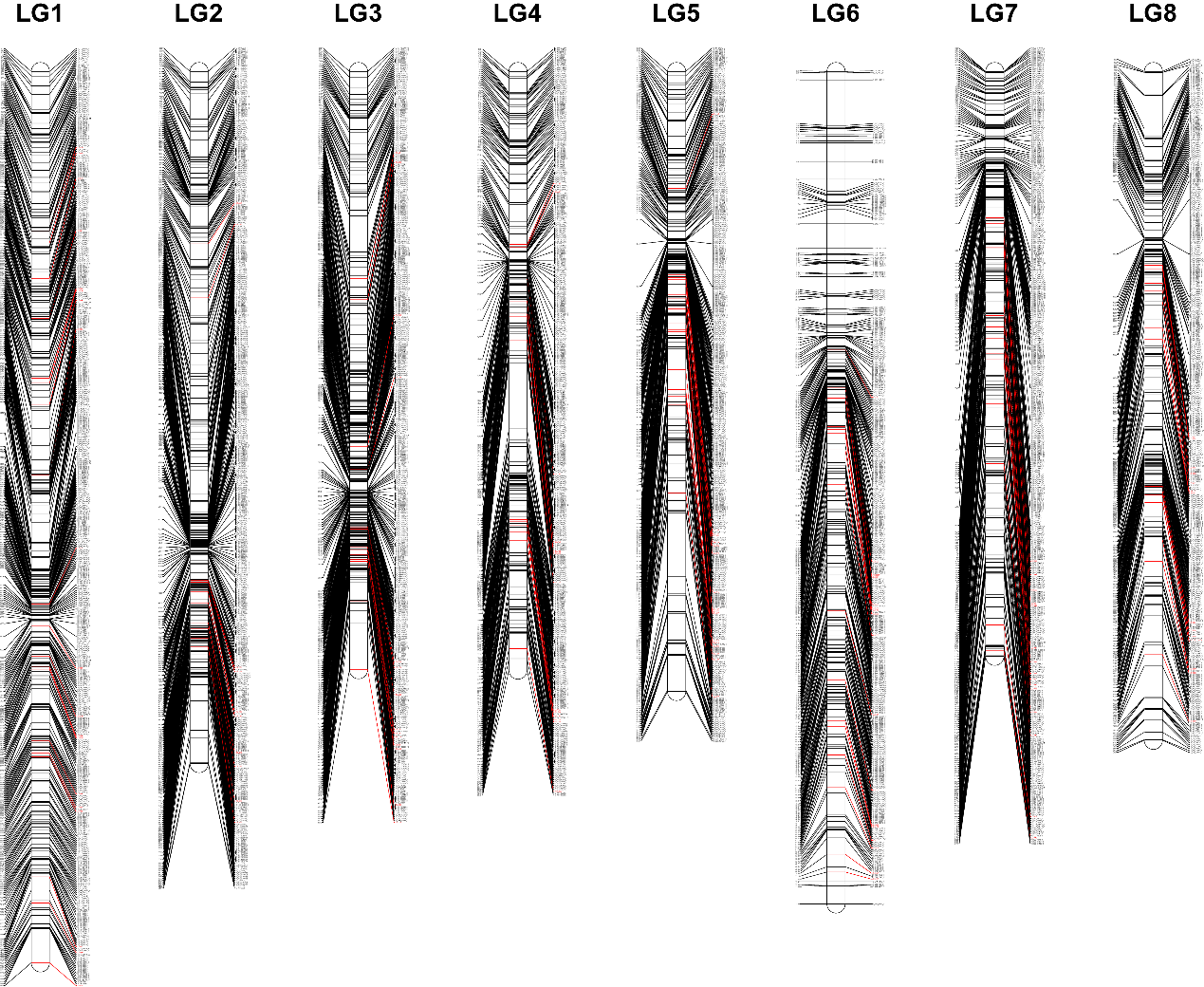
**

**Fig. S1 Integrated SSR-SNP linkage map**

118 SSR markers and MrFT2_BD-SEX marker were coloured in red.

**
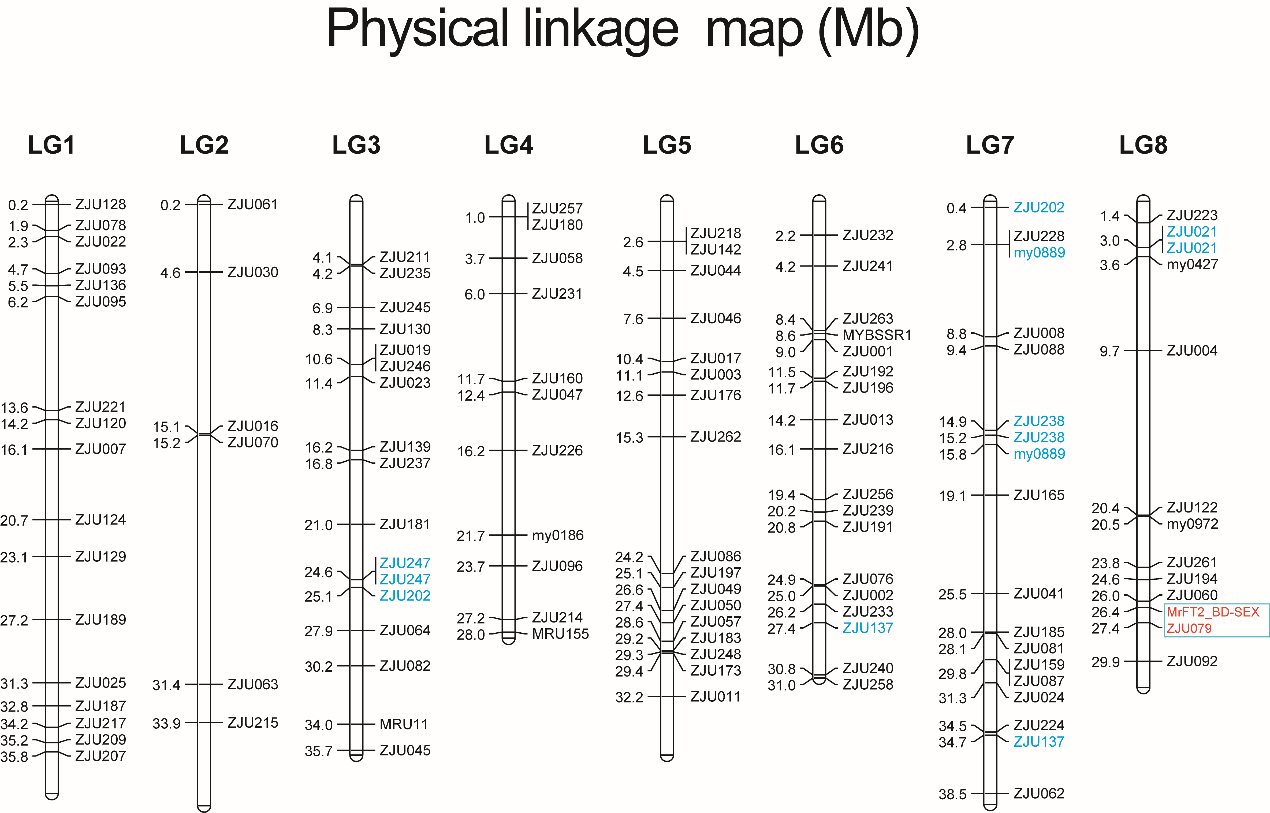
Fig. S2 Integrated SSR physical linkage map of red bayberry (*Morella rubra*).** Eight linkage groups (LG) consisting of 110 SSR markers and the MrFT2_BD-SEX. The markers were aligned to the genome physical map, in blue fonts are duplications. The boxed red markers were identified as being associated with sex trait.

**Tables**

**Table S1. Details of the 203 red bayberry accessions used in this study.**

♀: female individuals, ♂: male individuals, ♀ ♂: monoecious individuals. The 203 accessions belonging to subgroups are shown according to the previously population structure. The PCR amplification information of sex specific marker MrFT2_BD-SEX were shown.

--: not detected.

| Table S1. The information of 203 red bayberry accessions in this study. | | | | | | |
| --- | --- | --- | --- | --- | --- | --- |
| NO. | Accession | Sex | Origin | MrFT2_BD-SEX | | Subpopulation |
| 1 | Cimei | ♀ | Wenzhou, Zhejiang | 129 | 129 | Dongkui' female series |
| 2 | Dongkui | ♀ | Cixi, Zhejiang | 129 | 129 | Dongkui' female series |
| 3 | Heiruilin | ♀ | Taiwan | 129 | 129 | Dongkui' female series |
| 4 | HZ2012-2 | ♀ | Hangzhou, Zhejiang | 129 | 129 | Dongkui' female series |
| 5 | HZ2012-4 | ♀ | Hangzhou, Zhejiang | 129 | 129 | Dongkui' female series |
| 6 | Ruiguangmei | ♀ | Japan | 129 | 129 | Dongkui' female series |
| 7 | Wenlingbenmei | ♀ | Wenzhou, Zhejiang | 129 | 129 | Dongkui' female series |
| 8 | Zaoshuimei | ♀ | Jinhua, Zhejiang | 129 | 129 | Dongkui' female series |
| 9 | Chise | ♀ | Hangzhou, Zhejiang | 129 | 129 | Fenhong' female series |
| 10 | Fenhong | ♀ | Yuyao, Zhejiang | 129 | 129 | Fenhong' female series |
| 11 | Fenhong3 | ♀ | Shangyu, Zhejiang | 129 | 129 | Fenhong' female series |
| 12 | Jinqiantan | ♀ | Hangzhou, Zhejiang | 129 | 129 | Fenhong' female series |
| 13 | Shuijing | ♀ | Hangzhou, Zhejiang | 129 | 129 | Fenhong' female series |
| 14 | Shuijing1 | ♀ | Yuyao, Zhejiang | 129 | 129 | Fenhong' female series |
| 15 | Shuijing3 | ♀ | Shangyu, Zhejiang | 129 | 129 | Fenhong' female series |
| 16 | Xiazhihong | ♀ | Yuyao, Zhejiang | 129 | 129 | Fenhong' female series |
| 17 | Y2012-139 | ♀ | Yuyao, Zhejiang | 129 | 129 | Fenhong' female series |
| 18 | Y2012-145 | ♀ | Yuyao, Zhejiang | 129 | 129 | Fenhong' female series |
| 19 | Yangpingmei | ♀ | Taizhou, Zhejiang | 129 | 129 | Fenhong' female series |
| 20 | YG2012-73 | ♀ | Unkown | 129 | 129 | Fenhong' female series |
| 21 | YG2012-82 | ♀ | Unkown | 129 | 129 | Fenhong' female series |
| 22 | YG2012-85 | ♀ | Unkown | 129 | 129 | Fenhong' female series |
| 23 | Yuelipan2 | ♀ | Shangyu, Zhejiang | 129 | 129 | Fenhong' female series |
| 24 | YY2010-70 | ♀ | Yuyao, Zhejiang | 129 | 129 | Fenhong' female series |
| 25 | YY2010-72 | ♀ | Yuyao, Zhejiang | 129 | 129 | Fenhong' female series |
| 26 | YY2010-73 | ♀ | Yuyao, Zhejiang | 129 | 129 | Fenhong' female series |
| 27 | YY2010-76 | ♀ | Yuyao, Zhejiang | 129 | 129 | Fenhong' female series |
| 28 | Zaose | ♀ | Hangzhou, Zhejiang | 129 | 129 | Fenhong' female series |
| 29 | Guangdongdamei | ♀ | Guangzhou, Guangdong | 129 | 129 | Mixed sex subpop |
| 30 | Guangdongheimei | ♀ | Guangzhou, Guangdong | 129 | 129 | Mixed sex subpop |
| 31 | YG2012-105 | ♀ | Unkown | 129 | 129 | Mixed sex subpop |
| 32 | YG2012-113 | ♀ | Unkown | 129 | 129 | Mixed sex subpop |
| 33 | Dafudayexidi | ♀ | Suzhou, Jiangsu | 129 | 129 | Unstructured |
| 34 | Dahongpao | ♀ | Huzhou, Zhejiang | 129 | 129 | Unstructured |
| 35 | Daji | ♀ | Suzhou, Jiangsu | 129 | 129 | Unstructured |
| 36 | Dayeguang | ♀ | Hangzhou, Zhejiang | 129 | 129 | Unstructured |
| 37 | Fenhong2 | ♀ | Shangyu, Zhejiang | 129 | 129 | Unstructured |
| 38 | HZ2012-1 | ♀ | Hangzhou, Zhejiang | 129 | 129 | Unstructured |
| 39 | Liuyemei | ♀ | Jinhua, Zhejiang | 129 | 129 | Unstructured |
| 40 | Muyemei | ♀ | Jinhua, Zhejiang | 129 | 129 | Unstructured |
| 41 | Niuyemei | ♀ | Jingzhou,Hunan | 129 | 129 | Unstructured |
| 42 | Shangchongmei | ♀ | Jingzhou,Hunan | 129 | 129 | Unstructured |
| 43 | Tongzimei | ♀ | Jingzhou,Hunan | 129 | 129 | Unstructured |
| 44 | Anhaipianzaosheng | ♀ | Anhai, Fujian | 151 | 151 | Biqi' female series |
| 45 | Biqi | ♀ | Yuyao, Zhejiang | 151 | 151 | Biqi' female series |
| 46 | Biqi1 | ♀ | Yuyao, Zhejiang | 151 | 151 | Biqi' female series |
| 47 | Biqi2 | ♀ | Yuyao, Zhejiang | 151 | 151 | Biqi' female series |
| 48 | Biqi3 | ♀ | Yuyao, Zhejiang | 151 | 151 | Biqi' female series |
| 49 | Biqi4 | ♀ | Yuyao, Zhejiang | 151 | 151 | Biqi' female series |
| 50 | Biqi5 | ♀ | Yuyao, Zhejiang | 151 | 151 | Biqi' female series |
| 51 | HZ2012-11 | ♀ | Hangzhou, Zhejiang | 151 | 151 | Biqi' female series |
| 52 | HZ2012-9 | ♀ | Hangzhou, Zhejiang | 151 | 151 | Biqi' female series |
| 53 | Ningboyong56 | ♀ | Yuyao, Zhejiang | 151 | 151 | Biqi' female series |
| 54 | Sanjiaaowumei | ♀ | Ningbo, Zhejiang | 151 | 151 | Biqi' female series |
| 55 | Yangmeiwang | ♀ | Yuyao, Zhejiang | 151 | 151 | Biqi' female series |
| 56 | YG2012-106 | ♀ | Unkown | 151 | 151 | Biqi' female series |
| 57 | YG2012-116 | ♀ | Unkown | 151 | 151 | Biqi' female series |
| 58 | YG2012-118 | ♀ | Unkown | 151 | 151 | Biqi' female series |
| 59 | YG2012-125 | ♀ | Unkown | 151 | 151 | Biqi' female series |
| 60 | YG2012-21 | ♀ | Yuyao, Zhejiang | 151 | 151 | Biqi' female series |
| 61 | YG2012-30 | ♀ | Unkown | 151 | 151 | Biqi' female series |
| 62 | YG2012-35 | ♀ | Unkown | 151 | 151 | Biqi' female series |
| 63 | YG2012-44 | ♀ | Unkown | 151 | 151 | Biqi' female series |
| 64 | YG2012-47 | ♀ | Unkown | 151 | 151 | Biqi' female series |
| 65 | YG2012-51 | ♀ | Unkown | 151 | 151 | Biqi' female series |
| 66 | YG2012-54 | ♀ | Unkown | 151 | 151 | Biqi' female series |
| 67 | YG2012-67 | ♀ | Unkown | 151 | 151 | Biqi' female series |
| 68 | YG2012-70 | ♀ | Unkown | 151 | 151 | Biqi' female series |
| 69 | YG2012-71 | ♀ | Unkown | 151 | 151 | Biqi' female series |
| 70 | YG2012-80 | ♀ | Unkown | 151 | 151 | Biqi' female series |
| 71 | YG2012-81 | ♀ | Unkown | 151 | 151 | Biqi' female series |
| 72 | YG2012-95 | ♀ | Unkown | 151 | 151 | Biqi' female series |
| 73 | Yuelipan | ♀ | Yuyao, Zhejiang | 151 | 151 | Biqi' female series |
| 74 | Zaoqimimei | ♀ | Yuyao, Zhejiang | 151 | 151 | Biqi' female series |
| 75 | Zaoshenganmei | ♀ | Anhai, Fujian | 151 | 151 | Biqi' female series |
| 76 | Dingaomei | ♀ | Wenzhou, Zhejiang | 151 | 151 | Mixed sex subpop |
| 77 | Huangyemei | ♀ | Wenzhou, Zhejiang | 151 | 151 | Zhejiang male subpop |
| 78 | Dayegaozhuang | ♀ | Wenzhou, Zhejiang | 151 | 151 | Unstructured |
| 79 | HZ2011-11 | ♀ | Hangzhou, Zhejiang | 151 | 151 | Unstructured |
| 80 | Jianmei | ♀ | Cixi, Zhejiang | 151 | 151 | Unstructured |
| 81 | Putaoli | ♀ | Hangzhou, Zhejiang | 151 | 151 | Unstructured |
| 82 | Songjiang | ♀ | Cixi, Zhejiang | 151 | 151 | Unstructured |
| 83 | Songmaoli | ♀ | Hangzhou, Zhejiang | 151 | 151 | Unstructured |
| 84 | Tuda | ♀ | Wenzhou, Zhejiang | 151 | 151 | Unstructured |
| 85 | Tumei | ♀ | Wenzhou, Zhejiang | 151 | 151 | Unstructured |
| 86 | Wanqimimei | ♀ | Yuyao, Zhejiang | 151 | 151 | Unstructured |
| 87 | Xianghong | ♀ | Hangzhou, Zhejiang | 151 | 151 | Unstructured |
| 88 | YG2012-24 | ♀ | Unkown | 151 | 151 | Unstructured |
| 89 | YG2012-42 | ♀ | Unkown | 151 | 151 | Unstructured |
| 90 | YG2012-45 | ♀ | Unkown | 151 | 151 | Unstructured |
| 91 | YG2012-88 | ♀ | Unkown | 151 | 151 | Unstructured |
| 92 | Dayeshangchun | ♀ | Jingzhou,Hunan | 129 | 129 | New accessions |
| 93 | Mudongshangchun | ♀ | Jingzhou,Hunan | 129 | 129 | New accessions |
| 94 | Xiaoyeshangchun | ♀ | Jingzhou,Hunan | 129 | 129 | New accessions |
| 95 | Y2015-6 | ♀ | Jingzhou,Hunan | 151 | 151 | New accessions |
| 96 | C2010-4 | ♀ ♂ | Cixi, Zhejiang | 151 | 151 | Unstructured |
| 97 | Y2010-16 | ♂ | Yuyao, Zhejiang | -- | -- | Biqi' female series |
| 98 | Y2012-1 | ♂ | Yuyao, Zhejiang | -- | -- | Biqi' female series |
| 99 | Y2012-2 | ♂ | Yuyao, Zhejiang | -- | -- | Biqi' female series |
| 100 | Y2012-151 | ♂ | Shangyu, Zhejiang | -- | -- | Fenhong' female series |
| 101 | C2010-1 | ♂ | Cixi, Zhejiang | -- | -- | Zhejiang male subpop |
| 102 | C2010-15 | ♂ | Cixi, Zhejiang | -- | -- | Zhejiang male subpop |
| 103 | C2010-19 | ♂ | Cixi, Zhejiang | -- | -- | Zhejiang male subpop |
| 104 | C2010-20 | ♂ | Cixi, Zhejiang | -- | -- | Zhejiang male subpop |
| 105 | C2010-23 | ♂ | Cixi, Zhejiang | -- | -- | Zhejiang male subpop |
| 106 | C2010-28 | ♂ | Cixi, Zhejiang | -- | -- | Zhejiang male subpop |
| 107 | C2010-29 | ♂ | Cixi, Zhejiang | -- | -- | Zhejiang male subpop |
| 108 | C2010-39 | ♂ | Cixi, Zhejiang | -- | -- | Zhejiang male subpop |
| 109 | C2010-41 | ♂ | Cixi, Zhejiang | -- | -- | Zhejiang male subpop |
| 110 | C2010-45 | ♂ | Cixi, Zhejiang | -- | -- | Zhejiang male subpop |
| 111 | C2010-48 | ♂ | Cixi, Zhejiang | -- | -- | Zhejiang male subpop |
| 112 | C2010-49 | ♂ | Cixi, Zhejiang | -- | -- | Zhejiang male subpop |
| 113 | C2010-50 | ♂ | Cixi, Zhejiang | -- | -- | Zhejiang male subpop |
| 114 | C2010-51 | ♂ | Cixi, Zhejiang | -- | -- | Zhejiang male subpop |
| 115 | C2010-52 | ♂ | Cixi, Zhejiang | -- | -- | Zhejiang male subpop |
| 116 | W2011-10 | ♂ | Wenzhou, Zhejiang | -- | -- | Zhejiang male subpop |
| 117 | W2011-2 | ♂ | Wenzhou, Zhejiang | -- | -- | Zhejiang male subpop |
| 118 | W2011-3 | ♂ | Wenzhou, Zhejiang | -- | -- | Zhejiang male subpop |
| 119 | W2011-4 | ♂ | Wenzhou, Zhejiang | -- | -- | Zhejiang male subpop |
| 120 | W2011-7 | ♂ | Wenzhou, Zhejiang | -- | -- | Zhejiang male subpop |
| 121 | W2011-9 | ♂ | Wenzhou, Zhejiang | -- | -- | Zhejiang male subpop |
| 122 | C2010-13 | ♂ | Cixi, Zhejiang | -- | -- | Mixed male subpop |
| 123 | C2010-14 | ♂ | Cixi, Zhejiang | -- | -- | Mixed male subpop |
| 124 | C2010-17 | ♂ | Cixi, Zhejiang | -- | -- | Mixed male subpop |
| 125 | C2010-18 | ♂ | Cixi, Zhejiang | -- | -- | Mixed male subpop |
| 126 | C2010-2 | ♂ | Cixi, Zhejiang | -- | -- | Mixed male subpop |
| 127 | C2010-22 | ♂ | Cixi, Zhejiang | -- | -- | Mixed male subpop |
| 128 | C2010-24 | ♂ | Cixi, Zhejiang | -- | -- | Mixed male subpop |
| 129 | C2010-30 | ♂ | Cixi, Zhejiang | -- | -- | Mixed male subpop |
| 130 | C2010-31 | ♂ | Cixi, Zhejiang | -- | -- | Mixed male subpop |
| 131 | C2010-34 | ♂ | Cixi, Zhejiang | -- | -- | Mixed male subpop |
| 132 | C2010-36 | ♂ | Cixi, Zhejiang | -- | -- | Mixed male subpop |
| 133 | C2010-37 | ♂ | Cixi, Zhejiang | -- | -- | Mixed male subpop |
| 134 | C2010-38 | ♂ | Cixi, Zhejiang | -- | -- | Mixed male subpop |
| 135 | C2010-40 | ♂ | Cixi, Zhejiang | -- | -- | Mixed male subpop |
| 136 | C2010-43 | ♂ | Cixi, Zhejiang | -- | -- | Mixed male subpop |
| 137 | C2010-54 | ♂ | Cixi, Zhejiang | -- | -- | Mixed male subpop |
| 138 | FJ2011-38 | ♂ | Fuan, Fujian | -- | -- | Mixed male subpop |
| 139 | FJ2011-39 | ♂ | Fuan, Fujian | -- | -- | Mixed male subpop |
| 140 | FJ2011-40 | ♂ | Fuan, Fujian | -- | -- | Mixed male subpop |
| 141 | FJ2011-43 | ♂ | Fuan, Fujian | -- | -- | Mixed male subpop |
| 142 | GX2011-20 | ♂ | Guilin, Guangxi | -- | -- | Mixed male subpop |
| 143 | GZ2011-32 | ♂ | Qiandongnan, Guizhou | -- | -- | Mixed male subpop |
| 144 | H2011-12 | ♂ | Hangzhou, Zhejiang | -- | -- | Mixed male subpop |
| 145 | H2011-13 | ♂ | Hangzhou, Zhejiang | -- | -- | Mixed male subpop |
| 146 | JS2011-14 | ♂ | Suzhou, Jiangsu | -- | -- | Mixed male subpop |
| 147 | JS2011-18 | ♂ | Suzhou, Jiangsu | -- | -- | Mixed male subpop |
| 148 | JS2011-19 | ♂ | Suzhou, Jiangsu | -- | -- | Mixed male subpop |
| 149 | T2011-29 | ♂ | Taizhou, Zhejiang | -- | -- | Mixed male subpop |
| 150 | Y2010-20 | ♂ | Yuyao, Zhejiang | -- | -- | Mixed male subpop |
| 151 | Y2010-3 | ♂ | Yuyao, Zhejiang | -- | -- | Mixed male subpop |
| 152 | Y2010-40 | ♂ | Yuyao, Zhejiang | -- | -- | Mixed male subpop |
| 153 | Y2010-41 | ♂ | Yuyao, Zhejiang | -- | -- | Mixed male subpop |
| 154 | Y2010-51 | ♂ | Yuyao, Zhejiang | -- | -- | Mixed male subpop |
| 155 | C2010-55 | ♂ | Cixi, Zhejiang | -- | -- | Mixed sex subpop |
| 156 | JS2011-16 | ♂ | Suzhou, Jiangsu | -- | -- | Mixed sex subpop |
| 157 | T2011-30 | ♂ | Taizhou, Zhejiang | -- | -- | Mixed sex subpop |
| 158 | W2011-1 | ♂ | Wenzhou, Zhejiang | -- | -- | Mixed sex subpop |
| 159 | W2011-5 | ♂ | Wenzhou, Zhejiang | -- | -- | Mixed sex subpop |
| 160 | C2010-11 | ♂ | Cixi, Zhejiang | -- | -- | Unstructured |
| 161 | C2010-25 | ♂ | Cixi, Zhejiang | -- | -- | Unstructured |
| 162 | C2010-26 | ♂ | Cixi, Zhejiang | -- | -- | Unstructured |
| 163 | C2010-27 | ♂ | Cixi, Zhejiang | -- | -- | Unstructured |
| 164 | C2010-32 | ♂ | Cixi, Zhejiang | -- | -- | Unstructured |
| 165 | C2010-33 | ♂ | Cixi, Zhejiang | -- | -- | Unstructured |
| 166 | C2010-35 | ♂ | Cixi, Zhejiang | -- | -- | Unstructured |
| 167 | C2010-42 | ♂ | Cixi, Zhejiang | -- | -- | Unstructured |
| 168 | C2010-44 | ♂ | Cixi, Zhejiang | -- | -- | Unstructured |
| 169 | C2010-46 | ♂ | Cixi, Zhejiang | -- | -- | Unstructured |
| 170 | C2010-47 | ♂ | Cixi, Zhejiang | -- | -- | Unstructured |
| 171 | C2010-53 | ♂ | Cixi, Zhejiang | -- | -- | Unstructured |
| 172 | FJ2011-41 | ♂ | Fuan, Fujian | -- | -- | Unstructured |
| 173 | GX2011-23 | ♂ | Guilin, Guangxi | -- | -- | Unstructured |
| 174 | GX2011-24 | ♂ | Guilin, Guangxi | -- | -- | Unstructured |
| 175 | GZ2011-33 | ♂ | Qiandongnan, Guizhou | -- | -- | Unstructured |
| 176 | JS2011-15 | ♂ | Suzhou, Jiangsu | -- | -- | Unstructured |
| 177 | T2011-26 | ♂ | Taizhou, Zhejiang | -- | -- | Unstructured |
| 178 | T2011-31 | ♂ | Taizhou, Zhejiang | -- | -- | Unstructured |
| 179 | W2011-6 | ♂ | Wenzhou, Zhejiang | -- | -- | Unstructured |
| 180 | W2011-8 | ♂ | Wenzhou, Zhejiang | -- | -- | Unstructured |
| 181 | Y2010-1 | ♂ | Yuyao, Zhejiang | -- | -- | Unstructured |
| 182 | Y2010-11 | ♂ | Yuyao, Zhejiang | -- | -- | Unstructured |
| 183 | Y2010-12 | ♂ | Yuyao, Zhejiang | -- | -- | Unstructured |
| 184 | Y2010-13 | ♂ | Yuyao, Zhejiang | -- | -- | Unstructured |
| 185 | Y2010-14 | ♂ | Yuyao, Zhejiang | -- | -- | Unstructured |
| 186 | Y2010-15 | ♂ | Yuyao, Zhejiang | -- | -- | Unstructured |
| 187 | Y2010-17 | ♂ | Yuyao, Zhejiang | -- | -- | Unstructured |
| 188 | Y2010-18 | ♂ | Yuyao, Zhejiang | -- | -- | Unstructured |
| 189 | Y2010-19 | ♂ | Yuyao, Zhejiang | -- | -- | Unstructured |
| 190 | Y2010-21 | ♂ | Yuyao, Zhejiang | -- | -- | Unstructured |
| 191 | Y2010-22 | ♂ | Yuyao, Zhejiang | -- | -- | Unstructured |
| 192 | Y2010-23 | ♂ | Yuyao, Zhejiang | -- | -- | Unstructured |
| 193 | Y2010-4 | ♂ | Yuyao, Zhejiang | -- | -- | Unstructured |
| 194 | Y2010-7 | ♂ | Yuyao, Zhejiang | -- | -- | Unstructured |
| 195 | Y2010-9 | ♂ | Yuyao, Zhejiang | -- | -- | Unstructured |
| 196 | Y2015-1 | ♂ | Jingzhou,Hunan | -- | -- | New accessions |
| 197 | Y2015-2 | ♂ | Jingzhou,Hunan | -- | -- | New accessions |
| 198 | Y2015-3 | ♂ | Jingzhou,Hunan | -- | -- | New accessions |
| 199 | Y2015-4 | ♂ | Jingzhou,Hunan | -- | -- | New accessions |
| 200 | Y2015-5 | ♂ | Jingzhou,Hunan | -- | -- | New accessions |
| 201 | Y2015-7 | ♂ | Jingzhou,Hunan | -- | -- | New accessions |
| 202 | Y2015-8 | ♂ | Jingzhou,Hunan | -- | -- | New accessions |
| 203 | Y2015-9 | ♂ | Jingzhou,Hunan | -- | -- | New accessions |

**Table S2. Allele frequencies and genotype frequencies of 11 potential sex-associated markers in female and male populations.**

In this study, only five allele frequencies, which had highest value in female population or male population or the difference value between female and male, are shown in the table. The four highest genotype frequencies of SSR markers among female and male populations are shown. The frequencies of alleles and genotype are in red.

|  |  | Allele frequencies | | | | | Main Genotype data (genotype frequencies) | | | |
| --- | --- | --- | --- | --- | --- | --- | --- | --- | --- | --- |
| **my0427** | **Allele** | **227** | **229** | **231** | **235** |  |  |  |  |  |
|  | **male** | 0.350 | 0.196 | 0.042 | 0.159 |  | **227/227 (0.21)** | **227/229 (0.09)** | **227/235 (0.09)** |  |
|  | **female** | 0.026 | 0.447 | 0.174 | 0.042 |  | **229/231 (0.24)** | **229/229 (0.18)** |  |  |
| **ZJU021** | **Allele** | **204** | **206** | **208** | **212** | **214** |  |  |  |  |
|  | **male** | 0.012 | 0.165 | 0.549 | 0.006 | 0.122 | **208/208 (0.50)** | **206/206 (0.13)** |  |  |
|  | **female** | 0.113 | 0.425 | 0.188 | 0.161 | 0.000 | **206/212 (0.23)** | **206/206 (0.22)** | **204/208 (0.15)** |  |
| **my0972** | **Allele** | **217** | **219** | **221** | **223** | **233** |  |  |  |  |
|  | **male** | 0.189 | 0.151 | 0.269 | 0.085 | 0.005 | **221/221 (0.17)** | **217/221 (0.09)** |  |  |
|  | **female** | 0.021 | 0.218 | 0.090 | 0.287 | 0.186 | **219/223 (0.21)** | **223/233 (0.17)** |  |  |
| **ZJU060** | **Allele** | **225** | **233** | **241** | **243** |  |  |  |  |  |
|  | **male** | 0.019 | 0.156 | 0.118 | 0.297 |  | **243/243 (0.25)** | **233/233 (0.15)** | **241/241 (0.09)** |  |
|  | **female** | 0.111 | 0.000 | 0.479 | 0.179 |  | **241/241 (0.29)** | **223/241 (0.17)** | **225/243 (0.16)** |  |
| **MrFT2_BD-SEX** | **Allele** | **129** | **151** |  |  |  |  |  |  |  |
|  | **male** | NA | NA |  |  |  | **NA** |  |  |  |
|  | **female** | 0.484 | 0.516 |  |  |  | **151/null (0.52)** | **129/null (0.48)** |  |  |
| **ZJU079** | **Allele** | **124** | **128** | **130** | **132** | **134** |  |  |  |  |
|  | **male** | 0.253 | 0.098 | 0.294 | 0.088 | 0.041 | **124/130 (0.21)** | **120/130 (0.20)** | **124/132 (0.09)** | **124/132 (0.03)** |
|  | **female** | 0.192 | 0.242 | 0.027 | 0.176 | 0.258 | **124/128 (0.15)** | **128/134 (0.13)** | **124/132 (0.08)** | **128/132 (0.09)** |

**Table S3. The F1 seedlings used for sex specific markers PCR amplification and sex classification.**

The F1 that have female inflorescence are coloured in red, while the blue color indicates male hybrids.

| Table S3. The F1 seedlings used for sex specific markers PCR amplification and sex classification. | | | | | | |
| --- | --- | --- | --- | --- | --- | --- |
| Individuals |  | Sex | MrFT2_BD-SEX | ZJU079 | ZJU254 | Sex classification |
| Parents | Biqi | Female | 151/151 | 124/128 | 195/201 |  |
|  | Dongkui | Female/Male^a^ | 129/129 | 124/132 | 197/197 |  |
| F1 Progeny | BD11-1 | Female | 151/151 | 124/128 | 197/201 | Biqi series female |
|  | BD11-2 | Male | -- | 124/124 | 195/197 |  |
|  | BD11-3 | Male | -- | 124/124 | 195/197 |  |
|  | BD11-5 | Female | 151/151 | 124/128 | 197/201 | Biqi series female |
|  | BD11-6 | Female | 129/151 | 128/132 | 197/201 | Biqi and Dongkui series female |
|  | BD11-7 | Female | 129/151 | 128/132 | 197/201 | Biqi and Dongkui series female |
|  | BD11-9 | Female | 129/151 | 128/132 | 197/201 | Biqi and Dongkui series female |
|  | BD11-11 | Female | 129/151 | 128/132 | 197/201 | Biqi and Dongkui series female |
|  | BD11-13 | Male | -- | 124/124 | 195/197 |  |
|  | BD11-14 | Male | -- | 124/124 | 195/197 |  |
|  | BD11-15 | Female | 129/151 | 128/132 | 197/201 | Biqi and Dongkui series female |
|  | BD11-17 | Female | 151/151 | 124/128 | 197/201 | Biqi series female |
|  | BD11-18 | Male | -- | 124/124 | 195/197 |  |
|  | BD11-19 | Male | -- | 124/124 | 195/197 |  |
|  | BD11-22 | Female | 129/129 | 124/132 | 195/197 | Dongkui series female |
|  | BD11-25 | Female | 129/151 | 128/132 | 197/201 | Biqi and Dongkui series female |
|  | BD11-26 | Female | 151/151 | 124/128 | 197/201 | Biqi series female |
|  | BD11-27 | Male | -- | 124/124 | 195/197 |  |
|  | BD11-30 | Female | 129/129 | 124/132 | 195/197 | Dongkui series female |
|  | BD11-31 | Female | 129/129 | 124/132 | 195/197 | Dongkui series female |
|  | BD11-32 | Male | -- | 124/124 | 195/197 |  |
|  | BD11-34 | Female | 151/151 | 124/128 | 197/201 | Biqi series female |
|  | BD11-37 | Female | 129/129 | 124/124 | 195/197 | Dongkui series female |
|  | BD11-38 | Female | 129/151 | 128/132 | 197/201 | Biqi and Dongkui series female |
|  | BD11-39 | Female | 129/129 | 124/132 | 195/197 | Dongkui series female |
|  | BD11-41 | Male | -- | 124/124 | 195/197 |  |
|  | BD11-44 | Female | 129/151 | 128/132 | 197/201 | Biqi and Dongkui series female |
|  | BD11-46 | Female | 129/129 | 124/132 | 195/197 | Dongkui series female |
|  | BD11-47 | Female | 129/129 | 124/132 | 195/197 | Dongkui series female |
|  | BD11-49 | Female | 129/129 | 124/132 | 195/197 | Dongkui series female |
|  | BD11-50 | Female | 129/129 | 124/132 | 195/197 | Dongkui series female |
|  | BD11-51 | Female | 129/151 | 128/132 | 197/201 | Biqi and Dongkui series female |
|  | BD11-52 | Male | -- | 124/124 | 195/197 |  |
|  | BD11-56 | Female | 129/129 | 124/132 | 195/197 | Dongkui series female |
|  | BD11-57 | Female | 129/151 | 128/132 | 197/201 | Biqi and Dongkui series female |
|  | BD11-58 | Female | 151/151 | 124/128 | 197/201 | Biqi series female |
|  | BD11-60 | Female | 151/151 | 124/128 | 197/201 | Biqi series female |
|  | BD11-62 | Female | 151/151 | 124/128 | 197/201 | Biqi series female |
|  | BD11-63 | Male | -- | 124/124 | 195/197 |  |
|  | BD11-64 | Male | -- | 124/124 | 195/197 |  |
|  | BD11-65 | Female | 129/129 | 124/132 | 195/197 | Dongkui series female |
|  | BD11-66 | Female | 129/129 | 124/132 | 195/197 | Dongkui series female |
|  | BD11-67 | Female | 151/151 | 124/128 | 197/201 | Biqi series female |
|  | BD11-70 | Female | 151/151 | 124/128 | 197/201 | Biqi series female |
|  | BD11-71 | Female | 129/151 | 128/132 | 197/201 | Biqi and Dongkui series female |
|  | BD11-72 | Male | -- | 124/124 | 195/197 |  |
|  | BD11-73 | Female | 129/129 | 124/132 | 195/197 | Dongkui series female |
|  | BD11-74 | Female | 129/129 | 124/132 | 195/197 | Dongkui series female |
|  | BD11-76 | Female | 129/129 | 124/132 | 195/197 | Dongkui series female |
|  | BD11-79 | Female | 129/129 | 124/132 | 195/197 | Dongkui series female |
|  | BD11-80 | Female | 151/151 | 124/128 | 197/201 | Biqi series female |
|  | BD11-81 | Female | 129/129 | 124/132 | 195/197 | Dongkui series female |
|  | BD11-82 | Female | 151/151 | 128/132 | 197/201 | Biqi series female |
|  | BD11-84 | Female | 129/151 | 128/132 | 197/201 | Biqi and Dongkui series female |
|  | BD11-87 | Female | 129/151 | 128/132 | 197/201 | Biqi and Dongkui series female |
|  | BD11-88 | Female | 129/129 | 124/132 | 195/197 | Dongkui series female |
|  | BD11-91 | Female | 129/151 | 124/128 | 195/197 | Biqi and Dongkui series female |
|  | BD11-93 | Female | 151/151 | 124/128 | 197/201 | Biqi series female |
|  | BD11-94 | Male | -- | 124/124 | 195/197 |  |
|  | BD11-95 | Female | 151/151 | 128/132 | 197/201 | Biqi series female |
|  | BD11-96 | Female | 151/151 | 124/128 | 197/201 | Biqi series female |
|  | BD11-97 | Female | 129/129 | 124/132 | 195/197 | Dongkui series female |
|  | BD11-99 | Female | 129/129 | 124/132 | 195/197 | Dongkui series female |
|  | BD11-100 | Male | -- | 124/124 | 195/197 |  |
|  | BD11-101 | Female | 129/129 | 124/132 | 195/197 | Dongkui series female |
|  | BD11-103 | Female | 129/129 | 124/132 | 195/197 | Dongkui series female |
|  | BD11-104 | Female | 151/151 | 124/128 | 197/201 | Biqi series female |
|  | BD11-105 | Male | -- | 124/124 | 195/197 |  |
|  | BD11-107 | Female | 129/129 | 124/132 | 195/197 | Dongkui series female |
|  | BD11-109 | Female | 129/151 | 128/132 | 197/201 | Biqi and Dongkui series female |
|  | BD11-110 | Female | 129/129 | 124/132 | 195/197 | Dongkui series female |
|  | BD11-111 | Female | 129/129 | 124/132 | 195/197 | Dongkui series female |
|  | BD11-112 | Male | -- | 124/124 | 195/197 |  |
|  | BD11-113 | Female | 129/151 | 128/132 | 197/201 | Biqi and Dongkui series female |
|  | BD11-114 | Male | -- | 124/124 | 195/197 |  |
|  | BD11-115 | Male | -- | 124/124 | 195/197 |  |
|  | BD11-117 | Female | 129/129 | 124/124 | 195/197 | Dongkui series female |
|  | BD13-2 | Male | -- | 124/132 | 195/197 |  |
|  | BD13-3 | Female | 129/129 | 124/132 | 195/197 | Dongkui series female |
|  | BD13-9 | Male | -- | 124/124 | 195/197 |  |
|  | BD13-12 | Female | 129/129 | 124/132 | 195/197 | Dongkui series female |
|  | BD13-18 | Female | 129/129 | 124/132 | 195/197 | Dongkui series female |
|  | BD13-33 | Female | 151/151 | 124/128 | 197/201 | Biqi series female |
|  | BD13-46 | Male | -- | 124/124 | 195/197 |  |
|  | BD13-55 | Female | 151/151 | 124/128 | 197/201 | Biqi series female |
|  | BD13-70 | Male | -- | 124/124 | 195/197 |  |
|  | BD13-73 | Male | -- | 124/124 | 195/197 |  |
|  | BD13-78 | Female | 129/151 | 128/132 | 197/201 | Biqi and Dongkui series female |
|  | BD13-81 | Female | 129/151 | 128/132 | 197/201 | Biqi and Dongkui series female |
|  | BD13-83 | Male | -- | 124/124 | 195/197 |  |
|  | BD13-87 | Female | 129/129 | 124/132 | 195/197 | Dongkui series female |
|  | BD13-91 | Female | 129/129 | 124/132 | 195/197 | Dongkui series female |
|  | BD13-93 | Female | 129/129 | 128/132 | 197/201 | Dongkui series female |
|  | BD13-94 | Female | 129/151 | 128/132 | 197/201 | Biqi and Dongkui series female |
|  | BD13-106 | Female | 129/129 | 124/132 | 195/197 | Dongkui series female |

a: female cultivar that mutated branch bearing viable pollen

**Table S4. Characteristics of SSR markers in this study.**

| Marker | GenBank Accession No. | DDBJ Accession No. | Repeat motif | Reference | Link |
| --- | --- | --- | --- | --- | --- |
| MRU11 | - | - | (TA)9 | Zhang, S.M., Xu, C.J., Gao, Z.S. et al. Conserv Genet (2009) 10: 1605. | <https://doi.org/10.1007/s10592-008-9804-x> |
| MRU155 | - | - | (GA)8 |  |  |
| MYBSSR1 | - | - | (CT)13 |  |  |
| my0186 | - | AB239392 | (AG)11 | Terakawa M, Kikuchi S, Kanetani S, et al. Molecular Ecology Notes, 2010, 6(3):709-711. | <https://doi.org/10.1111/j.1471-8286.2006.01318.x> |
| my0427 | - | AB239393 | (CT)15 |  |  |
| my0793 | - | AB239397 | (CT)12 |  |  |
| my0889 | - | AB239400 | (AG)10 |  |  |
| my0972 | - | AB239401 | (CT)11 |  |  |
| ZJU001 | JQ318696 | - | (GA)10 | Jiao Y, Jia H.M., Li X, et al. Bmc Genomics, 2012, 13(1):201. | <https://doi.org/10.1186/1471-2164-13-201> |
| ZJU002 | JQ318697 | - | (TC)13 |  |  |
| ZJU003 | JQ318698 | - | (AG)11 |  |  |
| ZJU004 | JQ318699 | - | (GA)10 |  |  |
| ZJU006 | JQ318701 | - | (GA)10 |  |  |
| ZJU007 | JQ318702 | - | (AG)13 |  |  |
| ZJU008 | JQ318703 | - | (CT)10 |  |  |
| ZJU011 | JQ318706 | - | (GA)10 |  |  |
| ZJU013 | JQ318708 | - | (CT)10 |  |  |
| ZJU016 | JQ318711 | - | (TC)10 |  |  |
| ZJU017 | JQ318712 | - | (CT)13 |  |  |
| ZJU019 | JQ318714 | - | (GA)12 |  |  |
| ZJU021 | JQ318716 | - | (TG)10 |  |  |
| ZJU022 | JQ318717 | - | (GA)10 |  |  |
| ZJU023 | JQ318718 | - | (AG)15 |  |  |
| ZJU024 | JQ318719 | - | (TC)10 |  |  |
| ZJU025 | JQ318720 | - | (TC)10 |  |  |
| ZJU030 | JQ318725 | - | (CA)13 |  |  |
| ZJU041 | JQ318736 | - | (TC)11 |  |  |
| ZJU044 | JQ318739 | - | (GA)12 |  |  |
| ZJU045 | JQ318740 | - | (CT)10 |  |  |
| ZJU046 | JQ318741 | - | (AG)10 |  |  |
| ZJU047 | JQ318742 | - | (GA)13 |  |  |
| ZJU048 | JQ318743 | - | (CT)14 |  |  |
| ZJU049 | JQ318744 | - | (GAA)8 |  |  |
| ZJU050 | JQ318745 | - | (AG)11 |  |  |
| ZJU057 | JQ318752 | - | (CT)10 |  |  |
| ZJU058 | JQ318753 | - | (GT)10 |  |  |
| ZJU059 | JQ318754 | - | (TC)14 |  |  |
| ZJU060 | JQ318755 | - | (GT)8(GA)9 |  |  |
| ZJU061 | JQ318756 | - | (TC)11 |  |  |
| ZJU062 | JQ318757 | - | (TC)10 |  |  |
| ZJU063 | JQ318758 | - | (TC)12 |  |  |
| ZJU064 | JQ318759 | - | (GA)10 |  |  |
| ZJU070 | JQ318765 | - | (CT)11 |  |  |
| ZJU076 | JQ318771 | - | (AG)9 |  |  |
| ZJU078 | JQ318773 | - | (TC)10 |  |  |
| ZJU079 | JQ318774 | - | (TC)13 |  |  |
| ZJU081 | JQ318776 | - | (GA)8 |  |  |
| ZJU082 | JQ318777 | - | (CT)10 |  |  |
| ZJU084 | JQ318779 | - | (AG)9 |  |  |
| ZJU086 | JQ318781 | - | (TC)10 |  |  |
| ZJU087 | JQ318782 | - | (GA)9 |  |  |
| ZJU088 | JQ318783 | - | (CT)9 |  |  |
| ZJU089 | JQ318784 | - | (GA)8 |  |  |
| ZJU092 | JQ318787 | - | (TG)10 |  |  |
| ZJU093 | JQ318788 | - | (GA)10 |  |  |
| ZJU095 | JQ318790 | - | (AG)9 |  |  |
| ZJU096 | JQ318791 | - | (CT)10 |  |  |
| ZJU100 | JQ318795 | - | (TC)9 |  |  |
| ZJU120 | JQ318815 | - | (GA)8 |  |  |
| ZJU122 | JQ318817 | - | (TC)8 |  |  |
| ZJU124 | JQ318819 | - | (CT)10 |  |  |
| ZJU128 | JQ318823 | - | (AG)14 |  |  |
| ZJU129 | JQ318824 | - | (CT)10 |  |  |
| ZJU130 | JQ318825 | - | (GA)8 |  |  |
| ZJU136 | JQ318831 | - | (GA)10 |  |  |
| ZJU137 | JQ318832 | - | (TC)8 |  |  |
| ZJU139 | JQ318834 | - | (GA)12 |  |  |
| ZJU142 | JQ318837 | - | (TC)13 |  |  |
| ZJU144 | JQ318839 | - | (AG)12 |  |  |
| ZJU150 | JQ318845 | - | (AG)10 |  |  |
| ZJU159 | KF914760 | - | (TC)11 | Jia H.M., Shen Y.T., Jiao Y, et al. Journal of Zhejiang Universityence B, 2014, 15(11):997-1005. | <https://doi.org/10.1631/jzus.B1400051> |
| ZJU160 | KF914761 | - | (AC)8 |  |  |
| ZJU165 | KF914766 | - | (CT)12 |  |  |
| ZJU173 | KF914774 | - | (AG)8 |  |  |
| ZJU175 | KF914776 | - | (AG)8 |  |  |
| ZJU176 | KF914777 | - | (CT)10 |  |  |
| ZJU180 | KF914781 | - | (TG)9 |  |  |
| ZJU181 | KF914782 | - | (GA)8 |  |  |
| ZJU183 | KF914784 | - | (GA)9 |  |  |
| ZJU185 | KF914786 | - | (CT)8 |  |  |
| ZJU187 | KF914788 | - | (CT)8 |  |  |
| ZJU188 | KF914789 | - | (CT)9 |  |  |
| ZJU189 | KF914790 | - | (TC)9 |  |  |
| ZJU191 | KF914792 | - | (CT)10 |  |  |
| ZJU192 | KF914793 | - | (CT)8 |  |  |
| ZJU193 | KF914794 | - | (AG)10 |  |  |
| ZJU194 | KF914795 | - | (TC)11 |  |  |
| ZJU196 | KF914797 | - | (CT)8 |  |  |
| ZJU197 | KF914798 | - | (AT)9 |  |  |
| ZJU200 | KF914801 | - | (TC)10 |  |  |
| ZJU201 | KF914802 | - | (CT)8 |  |  |
| ZJU202 | KF914803 | - | (TC)9 |  |  |
| ZJU207 | KF914808 | - | (TC)9 |  |  |
| ZJU208 | KF914809 | - | (CT)8 |  |  |
| ZJU209 | KF914810 | - | (AG)9 |  |  |
| ZJU211 | KF914812 | - | (AG)8 |  |  |
| ZJU214 | KF914815 | - | (TC)9 |  |  |
| ZJU215 | KF914816 | - | (GA)10 |  |  |
| ZJU216 | KF914817 | - | (GA)9 |  |  |
| ZJU217 | KF914818 | - | (TC)16 |  |  |
| ZJU218 | KF914819 | - | (GA)9 |  |  |
| ZJU221 | KF914822 | - | (GA)9 |  |  |
| ZJU223 | KF914824 | - | (CT)10 |  |  |
| ZJU224 | KF914825 | - | (AG)8 |  |  |
| ZJU226 | KF914827 | - | (TC)10 |  |  |
| ZJU228 | KF914829 | - | (CT)8 |  |  |
| ZJU231 | KF914832 | - | (TC)10 |  |  |
| ZJU232 | KF914833 | - | (CT)8 |  |  |
| ZJU233 | KF914834 | - | (CT)10 |  |  |
| ZJU235 | KF914836 | - | (AG)9 |  |  |
| ZJU236 | KF914837 | - | (GA)8 |  |  |
| ZJU237 | KF914838 | - | (GA)14 |  |  |
| ZJU238 | KF914839 | - | (AC)8 |  |  |
| ZJU239 | KF914840 | - | (AG)10 |  |  |
| ZJU240 | KF914841 | - | (CT)8 |  |  |
| ZJU241 | KF914842 | - | (CT)9 |  |  |
| ZJU245 | KF914846 | - | (AG)10 |  |  |
| ZJU246 | KF914847 | - | (TC)8 |  |  |
| ZJU247 | KF914848 | - | (GA)10 |  |  |
| ZJU248 | KF914849 | - | (TC)9 |  |  |
| ZJU254 | KF914855 | - | (AG)9 |  |  |
| ZJU256 | KF914857 | - | (AG)8 |  |  |
| ZJU257 | KF914858 | - | (AG)23 |  |  |
| ZJU258 | KF914859 | - | (TC)8 |  |  |
| ZJU261 | KF914862 | - | (CT)8 |  |  |
| ZJU262 | KF914863 | - | (GA)8 |  |  |
| ZJU263 | KF914864 | - | (TC)8 |  |  |
